# Supplementary material for: Body Composition, Anemia, and Kidney Function among Guatemalan Sugarcane Workers
Source: Nutrients. 2021 Nov 2;13(11):3928. doi: 10.3390/nu13113928 (PMC8621317; doi:10.3390/nu13113928)
Supplement: Supplementary file 1 [file nutrients-13-03928-s001.zip › nutrients-1395541-SI.pdf]

nutrients

MDPI

Pantaleon

Example weekly menu: Pantaleon

Monday	Tuesday	Wednesday	Thursday	Friday	Saturday	Sunday
**Breakfast**						
Scrambled eggs	Boiled plantain and eggs	Eggs with ejote	Refried beans with eggs	Scrambled eggs	Eggs with ejote	Fried eggs
Tomato and onion	Black beans	Salsa	Pepper sauce	Sausage	Black beans	Tomato and onion
Refried beans	Cream	Black beans	Rice	Black beans	Cream	Black beans
	Corn Tortillas	Cheese	Cream	Cheese	Corn Tortillas	Cream
Corn tortillas	Smoothie	Corn tortillas	Corn tortillas	Corn tortillas	Coffee	Corn tortillas
Coffee		Coffee	Smoothie	Coffee		Smoothie
**Lunch**						
Sausage	Hard boiled eggs	Beef strips	Spaghetti with sausage	Eggs with rice	Sausage	Roasted chicken
Rice	Black beans	Rice	Black beans	Black beans	Black beans	Black beans
Black beans	Tomato salsa	Black beans				
Corn tortillas	Corn tortillas	Corn tortillas	Corn tortillas	Corn tortillas	Corn tortillas	Corn tortillas
Fruit	Fruit	Fruit	Fruit	Fruit	Fruit	Fruit
Juice beverage	Juice beverage	Juice beverage	Juice beverage	Juice beverage	Juice beverage	Juice beverage
**Dinner**						
Sauteed pork	Roasted chicken	White beans	Chicken soup	Grilled chicken with salsa	Chicken with noodles	Beef soup
Black beans	Macaroni salad	Sauteed pork	Rice	Black beans	Black beans	Squash and carrots
Rice	Black beans	Rice		Rice		Rice
Corn tortillas	Corn tortillas	Corn tortillas	Corn tortillas	Corn tortillas	Corn tortillas	Corn tortillas
Coffee	Juice beverage	Juice beverage	Juice beverage	Smoothie	Juice beverage	Juice beverage
Hot salsa	Hot salsa	Hot salsa	Hot salsa	Hot salsa	Hot salsa	Hot salsa

Figure S1. Sample weekly menu from Pantaleon for Altiplano workers.

Table S1. Pilot analysis of energy intake and macronutrient values among 49 sugarcane cutters in Guatemala, January 2019.

On a sample of 49 workers, 24 Altiplano and 25 Zona, dietary recall indicated a 24-hr energy intake for Zona workers of was 3836 kcal v. 4185 kcal for Altiplano workers. While mean kcal intake values were not statistically significantly different between the groups (p=0.12), it is notable that, on average, in this subset of workers, Zona workers consumed approximately 350 kcal per day less than did Altiplano workers. Protein intake values differed between groups. Compared to Altiplano workers, Zona workers consumed less protein, both for relative percent of energy derived from protein and for total protein intake in grams per day. In terms of energy derived from protein, Zona workers were found to consume 14% of kcal, compared to 15% among Altiplano workers (p=0.02). Total daily protein intake was lower in Zona versus Altiplano workers by a mean difference of 22 grams per day (158 v. 136 grams per day, respectively, p=0.02).

**Macronutrient**	**Cohort (n=49)**	**Altiplano (n=24)**	**Zona (n=25)**	***p*-value**
		**Mean (SD)**		
Energy intake (kcal)	4007.0 (781.6)	4184.8 (619.7)	3836.4 (889.8)	0.12
Carbohydrate (% of kcal)	66.0 (4.5)	66.5 (3.9)	65.5 (5.0)	0.45
Fat (% of kcal)	18.5 (4.0)	17.9 (3.7)	19.1 (4.2)	0.27
Protein (% of kcal)	14.6 (1.7)	15.2 (1.0)	14.1 (2.0)	**0.02**
Protein (g)	147.1 (33.2)	158.3 (21.8)	136.4 (38.8)	**0.02**
Protein avb (g)	32.8 (11.7)	36.0 (8.7)	28.4 (14.1)	0.07
